# Impact of Lebanon’s Economic Crisis on Cancer Care Delivery: A Multicenter Cross-Sectional Study of Treatment Initiation Delays and Incomplete Therapy

**DOI:** 10.3390/curroncol33080478

**Published:** 2026-08-14

**Authors:** Mohamad Ali Hachem, Nadeen Zayour, Elie Daibess, Jacqueline Najjar, Elie Jean Karam, Solay Farhat, Zeinab Hammoud, Ghadir M. Nasreddine, Mohamad Abbass, Zeinab Sleiman, Maroun Sadek, Ahmad Ibrahim, Issam Chehade, Bassam Matar

**Affiliations:** 1 Department of Internal Medicine, Division of Hematology and Oncology, Faculty of Medical Sciences, Lebanese University, Beirut P.O. Box 14-6573, Lebanon; d.ali24t@gmail.com (M.A.H.); eliedaibess94@gmail.com (E.D.); jacqueline.y.najjar@gmail.com (J.N.); elie_k11@hotmail.com (E.J.K.); ghadir.nasreddine@hotmail.com (G.M.N.); muhammad.hani.abbas@gmail.com (M.A.); sleimanzouzou@gmail.com (Z.S.); 2 International Department, Gustave Roussy Cancer Campus, 94800 Villejuif, France; 3 Organized Research Unit, Al Zahraa Hospital University Medical Center, Beirut P.O. Box 90-361, Lebanon; nadeenzayour6@gmail.com; 4 Internal Medicine Department, Faculty of Medical Sciences, Lebanese University, Beirut P.O. Box 14-6573, Lebanon; solayfarhat@gmail.com (S.F.); zeinabham2000@gmail.com (Z.H.); 5 Department of Hematology and Oncology, Lebanese Hospital Geitaoui-University Medical Center, Beirut, Lebanon; marounhsadek@gmail.com; 6 Department of Hematology and Oncology, Makassed General Hospital, Beirut P.O. Box 11-6301, Lebanon; ahmad_o_ibrahim@hotmail.com; 7 Department of Hematology and Oncology, Rafik Hariri Hospital, Beirut, Lebanon; chehade_iss@hotmail.com; 8 Department of Hematology and Oncology, Al Zahraa Hospital University Medical Center, Beirut P.O. Box 90-361, Lebanon

**Keywords:** cancer, Lebanon, economic crisis, financial vulnerability, treatment delays, incomplete treatment, cancer care delivery, NCCN guideline adherence

## Abstract

**Background/Objectives**: In Lebanon, the economic crisis since 2019 has severely strained healthcare infrastructure, yet its impact on cancer treatment adherence remains incompletely characterized. This study assessed factors associated with treatment delay and regimen modification to identify barriers to optimal cancer care among Lebanese patients. **Methods**: This multicenter, cross-sectional study was conducted in five tertiary hospitals in Beirut, Lebanon, between 13 June 2024, and 27 June 2025. Demographic, clinical and treatment data were collected via a structured questionnaire. Bivariate and multivariable binary logistic regression analyses were performed to identify independent predictors of treatment delay (>2 weeks) and incomplete treatment regimens. Adjusted odds ratios (aORs) with 95% confidence intervals (CIs) are reported. **Results**: A total of 244 patients were enrolled in the study. The study population was predominantly female (59.0%) and aged ≥60 years (60.2%). Advanced disease was common, with 50.4% of patients presenting with stage IV disease. Financial vulnerability was widespread: 72.6% reported monthly household incomes below USD 1000, 88.1% reported a decline in income due to the economic crisis, and 39.3% relied on unstable income sources. Overall, 52.5% of patients experienced treatment initiation delays, and 9.0% received incomplete or dose-reduced regimens. On multivariable analysis, reliance on unfixed income independently predicted treatment delay (aOR 2.55, 95% CI 1.42–4.59; *p* = 0.002), lack of health insurance (aOR 1.91, 95% CI 1.08–3.39; *p* = 0.026) and pre-treatment surgical costs also increased the odds of delay (aOR 9.03, 95% CI 2.57–31.7; *p* = 0.001). For incomplete treatment, unfixed income remained an independent predictor (aOR 2.09, 95% CI 1.16–5.79; *p* = 0.015). A lack of insurance (aOR 5.62, 95% CI 1.22–25.9; *p* = 0.027) and high laboratory costs (>USD 150 per session) were also associated with incomplete regimens (aOR 1.62, 95% CI 1.02–2.57; *p* = 0.041). **Conclusions**: In Lebanon’s crisis context, financial instability was a key factor associated with deviations in cancer treatment. These findings highlight the need for strengthened insurance coverage and subsidization of diagnostic and treatment-related costs to ensure timely and continuous oncologic care.

## Simple Summary

Cancer treatment receipt is crucial for optimal clinical outcomes; however, treatment may be suspected to be delayed or modified when patients experience financial vulnerability. This study explored factors associated with treatment delays and incomplete treatment regimens to provide insight into the impact of Lebanon’s ongoing economic crisis on cancer patients receiving hospital treatment. Treatment initiation delays were common, whereas suboptimal treatment delivery was observed yet less common. Further analysis revealed that patient reliance on unstable financial sources, lack of insurance, and high treatment-related costs increased the odds of these events. These findings are particularly important, especially given the costly and prolonged nature of cancer care and the financial instability experienced in Lebanon.

## 1. Introduction

Cancer remains one of the foremost public health challenges of the twenty-first century. According to estimates from the GLOBOCAN 2022 global cancer statistics database of the International Agency for Research on Cancer (IARC), approximately 20 million new cancer cases and 9.7 million cancer-related deaths are recorded worldwide [1]. The burden is disproportionately concentrated in low- and middle-income countries (LMICs), where approximately 70% of cancer deaths occur [1]. Across the Middle East and North Africa (MENA) region, the incidence of cancer increased by 54.2% between 1990 and 2021, far exceeding the global average increase of 25.3% [2]. In Lebanon, GLOBOCAN 2022 reported 13,034 new cancer cases and 7307 cancer-related deaths, placing the country among those with the highest cancer incidence rates in the MENA region [3].

In addition to its biological complexity, cancer inflicts substantial financial and psychosocial burdens on patients and their families. Treatment entails direct medical costs, out-of-pocket expenditures, income loss, and other indirect expenses that collectively diminish patients’ quality of life (QOL) and psychological well-being [4,5]. Even in countries with universal healthcare systems, such as Sweden, Norway, and Denmark, patients may face catastrophic health expenditures, defined as healthcare-related payments that place a substantial financial burden on households and may require difficult trade-offs between medical care and basic living needs [6,7]. In LMICs, where health financing is fragile and insurance coverage remains incomplete, these burdens are profoundly amplified [8,9]. Systematic reviews have shown that more than 70% of cancer patients in low-income countries experience catastrophic health expenditures, a prevalence over five times higher than that reported in high-income settings [9]. Furthermore, a population-based study conducted in Western Washington, USA, among patients diagnosed with cancer between 1995 and 2009, identified severe financial hardship as an independent predictor of early cancer mortality, highlighting the role of financial vulnerability as a prognostic factor alongside tumor biology [4].

Lebanon exemplifies this challenge. Since late 2019, the country has experienced one of the most severe economic collapses recorded globally since the mid-nineteenth century, driven by a combination of long-standing structural economic weaknesses, a financial and banking crisis, high public debt, political instability, and the impact of the COVID-19 pandemic and the Beirut port explosion. During this period, the Lebanese pound lost more than 90% of its precrisis value and the gross domestic product (GDP) contracted by approximately 58% between 2019 and 2021 [10,11]. Multidimensional poverty nearly doubled during this period, and the proportion of households reporting deprivation of healthcare access rose from 9% to 33% [11]. The healthcare sector has been severely affected: essential medications have become unaffordable or unavailable, third-party payer structures have weakened, and historically, the Ministry of Public Health (MOPH)-subsidized cancer drug access program has been undermined [12,13]. Recent evidence reported treatment interruption rates of approximately 32% among Lebanese cancer patients, driven primarily by drug scarcity and insurance coverage failure, with many patients increasingly relying on out-of-pocket payments to maintain continuity of care [12]. The economic crisis has also increased the cost of certain cancer treatments by as much as 768% [14]. Consequently, access to advanced therapies, including immunotherapy, targeted therapy, and selected chemotherapy agents, has become increasingly restricted, especially among socioeconomically disadvantaged patients, rural residents, and those lacking comprehensive insurance coverage [4,15,16]. These barriers have forced patients to delay, modify, or discontinue potentially life-saving therapies, which threatens both patient outcomes and prognosis [4,5,7].

Despite these challenges, there is limited prospective evidence examining treatment disruptions and their associated financial and clinical determinants among cancer patients in Lebanon. Given the unique combination of a high cancer burden and an ongoing economic collapse, there is an urgent need for context-specific evidence to inform national cancer care policies, resource allocation, and patient support strategies [15]. In this context, the present study was designed to address this important evidence gap. Specifically, the study aimed to (1) characterize the sociodemographic, economic, and clinical profiles of cancer patients receiving care in five major oncology centers in Lebanon and (2) examine the prevalence and independent financial and clinical characteristics associated with treatment delays and incomplete treatment regimens.

## 2. Materials and Methods

### 2.1. Study Design and Setting

This is a cross-sectional analytical study conducted across five tertiary care hospitals in Lebanon: (1) Al Zahraa Hospital University Medical Center, (2) Lebanese Hospital Geitaoui–University Medical Center, (3) Makassed General Hospital, (4) Rafik Hariri University Hospital, and (5) Saint George Hospital University Medical Center. These centers are located in Beirut and were selected because they are major tertiary oncology referral hospitals that provide care to patients from different governorates across Lebanon, enabling the recruitment of diverse patient populations beyond the Beirut area. The study was conducted from 13 June 2024 to 27 June 2025.

### 2.2. Participants

Patients were considered eligible for inclusion if they were Lebanese adults of either sex, aged 18 years or older, and received active cancer treatment, including chemotherapy, radiotherapy, and/or other systemic anticancer therapies at one of the selected study centers. The exclusion criteria included pregnancy as a precautionary ethical measure and receipt of treatment exclusively with oral anticancer medications. Patients receiving exclusively oral anticancer therapy were excluded because the study was conducted through hospital-based recruitment. These patients are typically managed in the clinic setting and are less likely to attend hospital infusion units, making systematic recruitment during the study period impractical.

### 2.3. Variables

The primary outcomes were as follows: (1) treatment delay, defined as the initiation of treatment more than 2 weeks after diagnosis; and (2) incomplete treatment, defined as not receiving the prescribed regimen or receiving a reduced dose relative to the planned regimen. Because there is no universally accepted definition of treatment initiation delay across different cancer types, and published studies have used heterogeneous definitions and time intervals, a threshold of >2 weeks was selected to allow a standardized assessment across all cancers included in the study. This threshold was informed by a review of the available literature [17] and established through expert consensus among the attending physician investigators from the five participating oncology centers during the investigators’ meeting.

Independent variables: Sociodemographic characteristics (sex, age group, governorate, educational attainment, marital status, occupational status); economic variables (monthly household income, income source stability, effect of the economic crisis on household income, medical insurance status and type of coverage); clinical variables (cancer stage, time since diagnosis, genetic testing, pre-treatment surgery); and cost variables (laboratory test costs, pre-treatment surgical costs).

Age was categorized into 18–29, 30–59, and ≥60 years. Educational level was classified as below high school education (including illiterate, primary, and middle school) or high school and above. Monthly household income was categorized as <USD 500, USD 500–1000, and >USD 1000. Surgical costs were categorized as USD 1000–3000, USD 3000–10,000, and USD 10,000–30,000, with separate categories for procedures covered by insurance and for patients who did not undergo surgery. Laboratory test costs were categorized into USD 10–39, 40–69, 70–109, 110–149, and ≥150 based on a review of laboratory charges across the five participating centers before study initiation. Costs were recorded in U.S. dollars (USD), as healthcare services at the participating hospitals were routinely priced and billed in USD during the study period. When payment was made in Lebanese pounds (LBP), patients reported the equivalent value, which was based on the prevailing official exchange rate of approximately LBP 89,500 per USD, which remained stable throughout the study period.

Compliance with the National Comprehensive Cancer Network (NCCN) guidelines was assessed based on whether the prescribed treatment regimen was consistent with NCCN recommendations at the time of treatment initiation [18]. Incomplete treatment was defined separately as failure to deliver the full prescribed regimen, including reduction of the planned drug dose or omission of one or more drugs included in the protocol. Therefore, NCCN compliance reflects the appropriateness of the initial treatment plan, whereas incomplete treatment reflects subsequent deviations during treatment administration. NCCN guideline compliance was included as a potential explanatory variable for incomplete treatment; however, these variables were considered distinct because guideline concordance was determined on the basis of the planned regimen, while incomplete treatment was determined based on treatment delivery.

### 2.4. Data Sources and Collection Procedures

Data were collected via a structured questionnaire developed for the study. On 6 June 2024, prior to study initiation, an investigator meeting was held during which all site teams, composed of oncology fellows responsible for patient recruitment and data collection at the participating centers, underwent standardized training to ensure consistency across centers. The training covered the study protocol, eligibility criteria, recruitment procedures, informed consent process, and standardized administration of the questionnaire. Additionally, simulation training sessions were conducted by the study coordinator (N.Z.) with each data collector to standardize the data collection procedures and to ensure an ethical approach when interacting with patients during recruitment and data collection.

The questionnaire captured demographic information; socioeconomic status of the patient and household (including employment status, monthly household income, income sources, financial coping mechanisms, and insurance coverage); clinical data (cancer type, stage, and time since diagnosis); and treatment-related variables, including delays between diagnosis and treatment initiation; details of the treatment plan (modality or modalities); and reasons for incomplete, delayed, or reduced treatment regimens as well as compliance with NCCN guidelines [18].

### 2.5. Bias Control

Bias was minimized through several strategies. Standardized training and simulation sessions were conducted for all data collectors prior to study initiation to reduce interviewer bias and ensure consistency across centers. A structured questionnaire with standardized wording and administration procedures was used for all participants. To reduce recall bias, information was cross-checked with available clinical records whenever possible, including data on time since diagnosis, time between diagnosis and treatment initiation, treatment modality, and treatment dosage.

### 2.6. Sample Size

A convenience sampling method was used to recruit eligible participants who were attending one of the five hospitals during the study period. A preliminary target sample size was considered during the study planning phase. However, the final sample size was determined by the number of eligible patients available during the study period. All patients who met the inclusion criteria and were available at the time of data collection were invited to participate, resulting in a final sample of 244 participants.

### 2.7. Statistical Methods

Descriptive statistics were computed to characterize the distribution of the sample profile and sociodemographic variables. Categorical variables are summarized as counts (n) and percentages, while outcome variables (treatment delay and overall treatment receipt) are presented as frequencies and percentages. Bivariate associations between categorical predictors and outcomes were assessed via the chi-square test or Fisher’s exact test, as appropriate. Variables with *p* < 0.05 in the bivariate analysis and/or those considered clinically relevant a priori were considered for inclusion in the multivariable binary logistic regression models. Bivariate statistical significance was not solely required for inclusion. The treatment-delay model included sex, age, educational level, occupational status, household income-source stability, medical insurance status, and pre-treatment surgical cost. Age was analyzed as a continuous variable and expressed per one-year increase; age categories were used only for descriptive and bivariate analyses. Because only 22 incomplete-treatment events were observed, the number of covariate parameters in that model was constrained to reduce the risk of overfitting and unstable estimates. Adjusted odds ratios (aORs) with 95% confidence intervals (CIs) are reported, and statistical significance was set at *p* < 0.05. All analyses were performed via Stata SE version 10.

### 2.8. Ethical Considerations

Ethical approval for this study was obtained from the Institutional Review Board (IRB) of all five participating centers: Al Zahraa Hospital University Medical Center (Approval No. 22/2023); Lebanese Hospital Geitaoui (Approval No. 2024-IRB-03); Makassed General Hospital (Approval No. 230124); Rafik Hariri University Hospital (Approval No. 2024-0205); and Saint George Hospital University Medical Center (Approval No. IRB-REC/O/013-25/0925). The study was conducted in accordance with the principles of the Declaration of Helsinki. All participants were informed about the purpose of the study, its procedures, potential risks and benefits, and their right to refuse participation or withdraw at any time without any consequences. Written informed consent was obtained from all participants or their legal representatives prior to data collection. Confidentiality and anonymity were strictly maintained throughout the study. No personally identifiable information was collected, and access to the dataset was restricted to the authorized study coordinator (N.Z.). The data were stored securely and used exclusively for research purposes.

## 3. Results

### 3.1. Demographic and Socioeconomic Characteristics of the Study Population

A total of 244 participants were included in the study. Among these, 144 were female (59.0%), and 100 were male (41.0%). Most participants were aged 60 years or older (n = 147; 60.2%), followed by those aged 30–59 years (n = 88; 36.1%), while a small proportion were aged 18–29 years (n = 9; 3.7%). With respect to educational attainment, 147 participants (60.2%) had completed high school or university education, whereas 97 (39.8%) were illiterate or had primary or middle-level education. In terms of occupational status, 101 participants were employed or retired (41.4%), and 143 were unemployed or currently students (58.6%). Most participants resided outside Beirut (n = 170; 69.7%), whereas 74 lived in Beirut (30.3%). The majority were currently married (n = 181; 74.2%), with the remainder being single, divorced, or widowed (n = 63; 25.8%).

Household income ranged across categories, with 89 participants reporting a monthly income of 500–1000 USD (36.5%), 67 reporting an income above 1000 USD (27.4%), and 88 reporting an income below 500 USD per month (36.1%). A decrease in household income since the onset of the economic crisis in Lebanon was reported by 215 participants (88.1%), whereas income remained unaffected for 29 (11.9%). A total of 96 participants relied on unfixed income sources such as informal work, transfers, debts, donations, or loans (39.3%), whereas 171 reported fixed income sources, including salaries, savings, pensions, or business ownership (70.1%). More than half of the participants did not have medical insurance (n = 139; 57.0%), while 105 (43.0%) reported having insurance. Among insured participants (n = 105), coverage most commonly included both hospital fees and anticancer treatment (n = 72; 68.6%), followed by hospital fees only (n = 22; 20.9%), treatment only (n = 4; 3.8%), or no coverage (n = 7; 6.7%) (Table 1).

### 3.2. Clinical and Treatment-Related Characteristics of the Study Population

Among the 244 cancer patients included in the analysis, 66 (27.0%) had breast cancer, 37 (15.2%) had colorectal cancer, 36 (14.7%) had lung cancer, 19 (7.8%) had Hodgkin or non-Hodgkin lymphoma, and 18 (7.4%) had ovarian cancer. The remaining 68 patients (27.9%) were diagnosed with other cancer types. Advanced-stage disease predominated, with more than half of the patients diagnosed with stage IV disease (n = 123; 50.4%), followed by stage III disease (n = 54; 22.1%), stage II disease (n = 39; 16.0%), and stage I disease (n = 15; 6.2%), while 13 patients (5.3%) were not staged. The majority of patients had been diagnosed within the past year (n = 187; 76.6%), whereas 57 patients (23.4%) had been diagnosed more than one year prior to data collection.

Overall, a delay in treatment initiation ranging from more than 2 weeks to more than 12 weeks after the cancer diagnosis was reported by 128 patients (52.5%), whereas 116 patients (47.5%) reported no treatment delay. Among patients who experienced treatment delay, the most frequently reported reasons were pre-treatment surgery (n = 59, 46.1%), delays in imaging reports (n = 36, 28.1%), financial constraints (n = 35, 27.3%), and delays in laboratory results (n = 33, 25.8%). Other reported reasons included administrative delays related to guarantor approval (e.g., the Ministry of Public Health or insurance providers) (n = 15, 11.7%), unavailability of treatment medications or inability to obtain the full prescribed dose (n = 12, 9.4%), and political instability or safety concerns related to the 2024 conflict in Lebanon (n = 6, 4.7%). Patients reported more than one reason for treatment delay. Regarding treatment receipt, most patients received the full prescribed treatment regimen compliant with the NCCN guidelines (n = 222; 91.0%). However, 22 patients received an incomplete treatment regimen or a reduced drug dosage. The reported reasons among these patients included financial constraints (59.1%), treatment unavailability (36.4%), contraindications (18.2%), full-dose drug shortages (13.6%), and treatment-related complications (9.1%). More than one reason could be reported.

With respect to specific direct medical costs, the cost of laboratory tests was most commonly reported in the 70–109 USD range (n = 87; 35.7%), followed by costs of 150 USD and above (n = 70; 28.7%), 10–39 USD (n = 49; 20.1%), 40–69 USD (n = 34; 13.9%), and 110–149 USD (n = 4; 1.6%). Regarding indirect medical costs, genetic testing was not performed for the majority of patients (n = 169; 69.3%), whereas 75 patients (30.7%) reported undergoing genetic testing. Pre-treatment cancer surgery was performed in 97 patients (39.8%), whereas 147 patients (60.2%) did not undergo surgery prior to treatment. Among patients reporting surgical costs, 28 paid between 1000 and 3000 USD (11.5%), 18 paid 3000–10,000 USD (7.4%), and 11 patients paid 10,000–30,000 USD (4.5%); however, most patients did not pay for cancer surgery because it was either covered by insurance or not performed (n = 187; 76.6%) (Table 2).

### 3.3. Bivariate Analysis of Factors Associated with Treatment Delay and Incomplete Treatment

Sociodemographic factors: Sex was not significantly associated with treatment delay, with higher proportions of delay observed among females (55.5%) than among males (44.5%) (*p* = 0.237). Likewise, sex was not significantly associated with receiving an incomplete treatment regimen, although incomplete treatment was slightly more common among females (72.7%) than males (23.7%) (*p* = 0.170). Age was also not significantly associated with either treatment delay (*p* = 0.378) or incomplete treatment (*p* = 0.446). Patients who were illiterate or had a primary or middle school level of education were significantly more likely to receive an incomplete treatment regimen than those with higher education (77.3% vs. 22.7%, *p* < 0.001). However, no significant association was observed between educational level and treatment delay (*p* = 0.180). Occupational status was not significantly associated with treatment delay (*p* = 0.117) or incomplete regimens (*p* = 0.062).

Economic factors: Household income level was not significantly associated with either treatment delay (*p* = 0.120) or incomplete treatment (*p* = 0.225), although treatment delays appeared more frequently among patients reporting household incomes below 500 USD. Reported income reduction due to the economic crisis was not significantly associated with treatment delay (*p* = 0.631) or treatment completeness (*p* = 0.523). In contrast, relying on an unfixed income was significantly associated with both outcomes. Patients reporting an unfixed income were significantly more likely to experience treatment delay (49.2% vs. 50.8%, *p* = 0.001) and to receive an incomplete treatment regimen (68.2% vs. 31.8%, *p* = 0.004).

Insurance-related factors: A lack of medical insurance was strongly associated with both treatment delay and incomplete treatment. Uninsured patients were significantly more likely to experience treatment delays than insured patients were (65.6% vs. 34.4%, *p* < 0.001). Similarly, incomplete treatment regimens were markedly more common among uninsured patients than among insured patients (90.9% vs. 9.1%, *p* = 0.012). The type of insurance coverage was also associated with treatment completeness. Patients without insurance coverage had the highest proportion of incomplete treatment regimens, whereas those with hospital coverage and treatment coverage had the lowest proportion (*p* = 0.012). The association between insurance coverage type and treatment delay approached statistical significance (*p* = 0.051).

Clinical factors: Cancer stage was not significantly associated with treatment delay (*p* = 0.319) or incomplete treatment (*p* = 0.631). The time since cancer diagnosis was not associated with treatment delay (*p* = 0.785); however, it was significantly associated with treatment completeness. Patients diagnosed more than one year prior to study enrollment were significantly more likely to receive an incomplete treatment regimen than those diagnosed within the past year (4.5% vs. 95.5%, *p* = 0.029). Genetic testing was not significantly associated with either treatment delay (*p* = 0.461) or incomplete treatment (*p* = 0.712).

Treatment-related factors: Undergoing surgery prior to treatment initiation was significantly associated with treatment delay. Patients who underwent pre-treatment surgery were more likely to experience treatment delays than were those who did not (57.8% vs. 42.2%, *p* = 0.004). However, pre-treatment surgery was not significantly associated with incomplete treatment (*p* = 0.905). The cost of surgery prior to treatment was significantly associated with treatment delay, especially among patients reporting higher surgical costs (*p* < 0.001). Laboratory test costs were not significantly associated with treatment delay (*p* = 0.349), but higher laboratory costs (≥150 USD) were significantly associated with incomplete treatment regimens (*p* = 0.024). Adherence to the NCCN clinical practice guidelines was significantly associated with both delayed (*p* = 0.019) and incomplete treatment (*p* < 0.001) (Table 3).

### 3.4. Multivariable Analysis of Predictors of Treatment Initiation Delay

Table 4 presents the multivariable logistic regression for delay in treatment initiation. After adjustment for all covariates, three variables remained independently and significantly associated with treatment delay. Patients with unfixed household income had more than twice the odds of delay than those with fixed income (aOR 2.55, 95% CI 1.42–4.59; *p* = 0.002). A lack of medical insurance increased the odds of delay (aOR 1.91, 95% CI 1.08–3.39; *p* = 0.026). Pre-treatment surgical costs ≥ 1000 USD increased the odds of delay around ninefold compared with patients who did not undergo surgery or who were covered by insurance (aOR 9.03, 95% CI 2.57–31.7; *p* = 0.001) (Table 4).

### 3.5. Multivariable Analysis of Predictors of Incomplete Treatment

Regarding factors associated with incomplete regimens in the overall treatment, as shown in Table 5, regression revealed that the household income source and medical insurance status were significantly associated with treatment modification. Participants with an unfixed household income had significantly greater odds of receiving a reduced treatment dose or the absence of one of the required treatment drugs than did those with a fixed income (OR = 2.09, 95% CI: 1.16–5.79; *p* = 0.015). In addition, participants without medical insurance had markedly greater odds of treatment modification than those with insurance coverage did (OR = 5.62, 95% CI: 1.22–25.9; *p* = 0.027). In addition, participants who incurred laboratory costs of 150 USD per session or higher had significantly greater odds of incomplete treatment than those with lower laboratory costs did (OR = 1.62, 95% CI: 1.02–2.57; *p* = 0.041). Higher educational attainment had a borderline protective effect (aOR 0.33, 95% CI 0.10–1.07; *p* = 0.056) (Table 5).

## 4. Discussion

This multicenter cross-sectional study of 244 patients with cancer provides real-world data on the financial burden of cancer care in Lebanon during the country’s ongoing economic crisis. The study population exhibited substantial socioeconomic vulnerability, with most participants being unemployed, lacking medical insurance coverage, and reporting low household incomes, with some households earning monthly incomes below 500 USD. Furthermore, 215 patients reported a decline in household income as a direct consequence of the economic crisis, highlighting the widespread financial hardship experienced by the cohort. These findings are consistent with previous evidence from Lebanon. Ladner et al. reported that the Lebanese multidimensional poverty index nearly doubled between 2019 and 2021, whereas the proportion of households deprived of healthcare access increased from 9% to 33% during the same period [14].

One of the most notable findings of this study was the coexistence of a relatively high prevalence of treatment initiation delays (52.5%) with high adherence to NCCN-recommended treatment regimens among patients who eventually started treatment. This rate compares unfavorably with rates reported in neighboring and comparable LMIC settings. A systematic review by Brand et al. reported variable treatment intervals across LMICs and identified financial constraints and system-level barriers as primary contributors [19]. In Lebanon specifically, Eid et al. documented treatment interruption in 31.9% of patients, which was attributed primarily to drug shortages and insurance failures [12]. Our data suggest that treatment delay is even more prevalent, reflecting the extent to which economic strain is reshaping cancer care delivery and steering patients away from timely initiation of standard treatment. The reported reasons for delayed treatment initiation among the study patients included the need for surgery, delays in obtaining imaging results, financial constraints, logistical challenges, and drug unavailability. These findings highlight the complexity of cancer care pathways, which involve multiple steps that may become bottlenecks during periods of economic instability. Furthermore, the high prevalence of stage IV disease (50.4%) observed in our cohort is consistent with patterns reported in many LMICs, where financial barriers, limited access to screening programs, and delays in healthcare seeking contribute to diagnosis at advanced-stage presentation [19]. This predominance of advanced-stage disease, together with the high frequency of treatment delays, suggests that access barriers may occur before treatment initiation rather than during treatment planning and delivery, underscoring the importance of strengthening early cancer detection and timely access to oncologic care.

In our cohort, 39.3% of patients reported reliance on unfixed income sources rather than stable sources of income. Income instability is not only a marker of low socioeconomic status but also of financial unpredictability, which may be particularly detrimental in cancer care, where treatment regimens require consistent financial capacity. A strong association between unfixed income and both delayed treatment initiation and incomplete treatment was observed. Patients relying on sporadic income sources may postpone treatment while attempting to secure financial resources and may be less able to sustain the recurring costs associated with therapy administration, resulting in treatment delays and incomplete treatment patterns. These findings align with the meta-analysis by Kitaw et al., which identified income instability as a major determinant of catastrophic health expenditure among patients with cancer globally [9].

Approximately 57% of our cohort lacked any form of health insurance coverage. According to the multivariable analysis, the association between a lack of health insurance and incomplete treatment was striking in magnitude. Uninsured patients had 5.62-fold higher odds of receiving incomplete treatment. Health insurance likely mitigates financial barriers throughout cancer care by reducing out-of-pocket expenditures and improving access to essential treatments and services. In Lebanon, the importance of insurance coverage is further amplified by the ongoing economic and healthcare crisis. Since 2019, the MOPH’s historically subsidized cancer drug program has deteriorated substantially [12,13], while the cost of certain cancer treatments has increased by up to 768% [14]. In addition, a significant association between a lack of insurance and treatment delay was observed in our multivariable analysis. This finding is similar to that reported by Yabroff et al. [20]. This suggests that insurance status may influence treatment initiation in the Lebanese context, as some patients may struggle to find financial resources to cover their treatment. These findings highlight the need for strengthened healthcare financing mechanisms and expanded insurance coverage for vulnerable populations.

In addition to income instability and insurance status, specific out-of-pocket healthcare expenditures have emerged as important determinants of treatment outcomes. Higher surgical costs highly predicted treatment delay, whereas higher laboratory costs were associated with incomplete treatment. Surgical procedures often represent substantial upfront expenses that may exhaust available financial reserves before systemic cancer treatment begins [9,21]. Similarly, although laboratory investigations are individually less costly than cancer therapies are, repeated monitoring throughout treatment can accumulate into a considerable financial burden. These findings suggest that financial toxicity extends beyond the cost of anticancer drugs and encompasses the broader diagnostic and monitoring services required for effective cancer care.

The relatively low proportion of patients who underwent genetic testing may be attributed to several factors. First, the high cost of genetic testing has been identified as a major barrier to access, particularly in LMICs, limiting its affordability and uptake among cancer patients [22,23,24]. This challenge is especially relevant in Lebanon, where ongoing economic difficulties have placed substantial financial burdens on patients and healthcare systems. Second, genetic testing is not universally indicated across all cancer types, stages, and clinical scenarios; therefore, many patients may not have met the criteria for testing according to current NCCN recommendations [18]. Furthermore, the limited patient awareness and understanding of genetic testing may have contributed to its low utilization. Previous studies have shown that oncology patients often report low confidence in their knowledge of genetic testing, may perceive it as irrelevant to their immediate care, and frequently prioritize more pressing concerns such as treatment affordability and out-of-pocket expenses [22]. While no significant association was observed between genetic testing and treatment-related outcomes in this study, expanding equitable access to genetic testing remains an important component of high-quality, personalized oncologic care.

Interestingly, household income and occupation were not significantly associated with either treatment delay or incomplete treatment. This finding suggests that financial vulnerability may be better captured by measures of economic security and healthcare affordability than by reported income alone. Contrary to expectations, cancer stage was not associated with treatment delay or incomplete treatment. Financial and healthcare access barriers may affect patients similarly regardless of disease stage during periods of severe economic hardship. Likewise, age and sex were not independently associated with either outcome. Although higher educational attainment demonstrated a borderline protective effect against incomplete treatment, this association did not reach statistical significance. This trend may reflect improved health literacy, better navigation of healthcare systems, and greater ability to access financial or social support mechanisms among more educated patients. However, in the context of Lebanon’s severe economic crisis, structural financial barriers may outweigh the protective effect of education, limiting its independent impact on treatment continuity. Collectively, these findings suggest that economic factors may exert a greater influence on treatment access and continuity than demographic or disease-related characteristics in this setting.

To the best of our knowledge, this study provides one of the few comprehensive assessments of barriers to timely and complete cancer treatment among Lebanese cancer patients in a country facing substantial economic constraints. The multicenter design, including five tertiary referral centers, enhances the diversity of the study population and improves the applicability of findings across major oncology care settings in the country. Data collection was performed using a structured questionnaire, complemented where possible by verification with clinical records, which helped improve data quality and reduce misclassification. In addition, the study simultaneously examined a range of sociodemographic, economic, and clinical variables, enabling a comprehensive assessment of the characteristics of treatment outcomes in a real-world clinical context. Finally, the use of multivariable regression analyses strengthened the validity of the findings by adjusting for potential confounding factors and enabling the identification of independent predictors of treatment delay and incomplete treatment.

Several limitations of this study warrant acknowledgment. First, the sample was drawn from five tertiary referral centers, and the sample size was smaller than initially anticipated because some patients did not meet the eligibility criteria. Consequently, the findings may not fully reflect the national burden of treatment delays and incomplete treatment, particularly among patients receiving care in smaller hospitals or underserved rural areas. Second, patients receiving exclusively oral anticancer therapy were excluded because of the hospital-based recruitment approach. This exclusion may have introduced selection bias, as patients treated with oral agents may have treatment costs and access to care different than patients receiving hospital-based therapy. Therefore, the results may not be fully generalizable to all patients with cancer. Third, the cost data were self-reported and may be subject to recall bias. In addition, cultural sensitivities surrounding discussions of debt, financial hardship, and the receipt of charitable assistance may have influenced responses to financial-related questions. Fourth, the relatively small number of incomplete treatment events (n = 22) may have reduced the statistical power and precision of analyses related to this outcome and increased the risk of overfitting in the multivariable logistic regression model. Given the limited number of outcome events, the number of covariates included in the model was restricted to maintain model stability; therefore, residual confounding by potentially relevant factors cannot be completely excluded. Age was included as a potential demographic confounder in the treatment-delay model but was not independently associated with treatment delay, and its inclusion did not materially alter the associations observed for the principal financial variables. In contrast, age was not included in the primary incomplete-treatment model because of the limited number of outcome events. Consequently, residual confounding by age cannot be excluded for this outcome. Although all eligible patients available during the study period were recruited, further extension of the recruitment period was not feasible. Therefore, the findings related to incomplete treatment should be interpreted with caution and validated in larger studies with a greater number of outcome events. Fifth, the cross-sectional design precludes the establishment of causal relationships between the examined factors and treatment outcomes. Sixth, despite adjustment for multiple covariates, residual confounding from unmeasured factors such as health literacy, social support, physician decision-making in intentionally delaying, modifying, or discontinuing treatment, and institutional practices, such as appointment availability, waiting times, access to medications, and financial assistance programs, cannot be excluded. Finally, this study did not include survival or quality-of-life outcomes, preventing direct assessment of the long-term clinical impact of treatment delays and incomplete treatment on patient prognosis.

## 5. Conclusions

Financial vulnerability, particularly unstable income sources, lack of insurance coverage, and out-of-pocket expenditures related to surgery and laboratory monitoring, represents a more important factor associated with treatment delay and incomplete treatment than traditional demographic or disease-related factors. This study highlights the profound impact of Lebanon’s economic crisis on cancer care delivery and underscores the need for policies aimed at strengthening financial protection mechanisms and reducing out-of-pocket healthcare expenditures among patients with cancer.

## Figures and Tables

**Table 1:** Demographic and socioeconomic characteristics of the study participants (n = 244).

Variable	n	%
**Sex**		
Female	144	59.0
Male	100	41.0
**Age Group (years)**		
18–29	9	3.7
30–59	88	36.1
≥60	147	60.2
**Educational attainment**		
Illiterate, primary, middle school education	97	39.8
Higher education	147	60.2
**Occupation**		
Employed or retired	101	41.4
Unemployed or student	143	58.6
**Governorate**		
Beirut	74	30.3
Other governorates	170	69.7
**Marital status**		
Married	181	74.2
Single, divorced, or widowed	63	25.8
**Household income (USD/month)**		
<500	88	36.1
500–1000	89	36.5
>1000	67	27.4
**Effect of economic crisis on household income**		
Household income unaffected	29	11.9
Household income decreased	215	88.1
Household income source *		
Fixed (salary, savings, pension, personal business)	171	70.1
Unfixed (informal, transfers, loans, aid)	96	39.3
**Medical insurance**		
No insurance	139	57.0
Insured	105	43.0
**Insurance coverage type (among insured, n = 105)**		
Hospital fees and treatment	72	68.6
Hospital fees only	22	20.9
Treatment only	4	3.8
No effective coverage	7	6.7

* Percentages may not sum to 100%, as participants could report multiple income sources.

**Table 2:** Clinical characteristics, treatment-related factors, and economic barriers among the study participants (N = 244).

Variable	n	%
**Current cancer diagnosis**		
Breast	66	27.0
Colorectal	37	15.2
Lung	36	14.7
Lymphoma	19	7.8
Ovarian	18	7.4
Other types	68	27.9
**Stage of cancer**		
Not staged	13	5.3
Stage I	15	6.2
Stage II	39	16.0
Stage III	54	22.1
Stage IV	123	50.4
**Time since cancer diagnosis**		
Less than 1 year	187	76.6
More than 1 year	57	23.4
**Treatment initiation elay (>2 weeks since diagnosis)**		
No delay	116	47.5
Delay present	128	52.5
Reasons for treatment delay (among delayed, n = 128) *		
Pre-treatment surgery	59	46.1
Delayed imaging reports	36	28.1
Financial constraints	35	27.3
Delayed laboratory results	33	25.8
Logistics (delay in guarantor approval)	15	11.7
Treatment unavailability or lack of full dose	12	9.4
Political instability and safety concerns	6	4.7
**Overall treatment received**		
Full prescribed regimen	222	91.0
Incomplete or reduced dose	22	9.0
**Reasons for incomplete treatment or reduced dose** (n = 22) **		
Financial constraints	13	59.1
Treatment unavailability	8	36.4
Contraindication	4	18.2
Full-dose drug shortage	3	13.6
Complication	2	9.1
**NCCN treatment compliance**		
Non-compliant	37	15.2
Compliant	207	84.8
**Direct medical costs-laboratory tests (USD/session)**		
10–39	49	20.1
40–69	34	13.9
70–109	87	35.7
110–149	4	1.6
≥150	70	28.7
**Indirect costs-genetic testing**		
Not performed	169	69.3
Performed	75	30.7
**Indirect costs-Pre-treatment surgery**		
Not performed	147	60.2
Performed	97	39.8
**Pre-treatment surgical cost (USD)**		
1000–3000	28	11.5
3000–10,000	18	7.4
10,000–30,000	11	4.5
Covered by insurance or not performed	187	76.6

* Multiple reasons possible; percentages calculated among the 128 patients with treatment delay. ** Multiple reasons possible; percentages calculated among the 22 patients with incomplete treatment or a reduced dose. NCCN = National Comprehensive Cancer Network.

**Table 3:** Bivariate analysis of factors associated with treatment delay and incomplete treatment.

Variable	Category	No Delay n (%)	Delay n (%)	*p*-Value (Delayed Initiation)	Complete Treatment n (%)	Incomplete Treatment n (%)	*p*-Value (Incomplete Treatment)
**SOCIODEMOGRAPHIC FACTORS**							
**Sex**	Female	73 (62.9)	71 (55.5)	0.237	128 (57.7)	16 (72.7)	0.170
	Male	43 (37.1)	57 (44.5)		94 (42.3)	6 (27.3)	
**Age group**	18–29 years	6 (5.2)	3 (2.3)	0.378	9 (4.1)	0 (0.0)	0.446
	30–59 years	44 (37.9)	44 (34.4)		78 (35.1)	10 (45.5)	
	≥60 years	66 (56.9)	81 (63.3)		135 (60.8)	12 (54.5)	
**Education**	<High school	41 (35.3)	56 (43.8)	0.180	80 (36.0)	17 (77.3)	**<0.001**
	High school or higher	75 (64.7)	72 (56.3)		142 (64.0)	5 (22.7)	
**Occupation**	Employed/retired	42 (36.2)	59 (46.1)	0.117	96 (43.2)	5 (22.7)	0.062
	Unemployed/student	74 (63.8)	69 (53.9)		126 (56.8)	17 (77.3)	
**ECONOMIC FACTORS**							
**Household** **income**	<500 USD	35 (30.2)	53 (41.4)	0.120	77 (34.7)	11 (50.0)	0.225
	500–1000 USD	49 (42.2)	40 (31.3)		81 (36.5)	8 (36.4)	
	>1000 USD	32 (27.6)	35 (27.3)		64 (28.8)	3 (13.6)	
**Income reduced by economic crisis**	Yes	101 (87.1)	114 (89.1)	0.631	183 (82.4)	19 (86.4)	0.523
	No	15 (12.9)	14 (10.9)		39 (17.6)	3 (13.6)	
**Income source**	Fixed	83 (71.6)	65 (50.8)	**0.001**	141 (63.5)	7 (31.8)	**0.004**
	Unfixed	33 (28.5)	63 (49.2)		81 (36.5)	15 (68.2)	
**MEDICAL INSURANCE FACTORS**							
**Insurance**	Insured	61 (52.6)	44 (34.4)	**<0.001**	103 (46.4)	2 (9.1)	**0.012**
	Uninsured	55 (47.4)	84 (65.6)		119 (53.6)	20 (90.9)	
**Coverage type**	Hospital + treatment	40 (34.5)	32 (25.0)	0.051 *	71 (32.0)	1 (4.6)	**0.012**
	Hospital only	15 (12.9)	7 (5.5)		22 (9.9)	0 (0.0)	
	Treatment only	2 (1.7)	2 (1.6)		4 (1.8)	0 (0.0)	
	No coverage	4 (3.5)	3 (2.3)		6 (2.7)	1 (4.6)	
	Uninsured	55 (47.4)	84 (65.6)		119 (53.6)	20 (90.9)	
**CLINICAL FACTORS**							
**Cancer stage**	Not staged	4 (3.5)	9 (7.0)	0.319	11 (5.0)	2 (9.1)	0.631
	Stage I	8 (6.9)	7 (5.5)		13 (5.9)	2 (9.1)	
	Stage II	18 (15.5)	21 (16.4)		34 (15.3)	5 (22.7)	
	Stage III	21 (18.1)	33 (25.8)		51 (23.0)	3 (13.6)	
	Stage IV	65 (56.0)	58 (45.3)		113 (50.9)	10 (45.5)	
**Time since diagnosis**	<1 year	88 (75.9)	99 (77.3)	0.785	166 (74.8)	21 (95.5)	**0.029**
	≥1 year	28 (24.1)	29 (22.7)		56 (25.2)	1 (4.5)	
**Genetic testing**	Not performed	83 (71.6)	86 (67.2)	0.461	153 (68.9)	16 (72.7)	0.712
	Performed	33 (28.4)	42 (32.8)		69 (31.1)	6 (27.3)	
**TREATMENT-RELATED FACTORS**							
**Pre-treatment surgery**	No	93 (80.2)	54 (42.2)	**0.004**	134 (60.4)	13 (59.0)	0.905
	Yes	23 (19.8)	74 (57.8)		88 (39.6)	9 (40.9)	
**Surgery cost**	1000–3000 USD	7 (6.0)	21 (16.4)	**<0.001**	22 (9.9)	6 (27.3)	0.073
	3000–10,000 USD	3 (2.6)	15 (11.7)		16 (7.2)	2 (9.1)	
	10,000–30,000 USD	0 (0.0)	11 (8.6)		11 (5.0)	0 (0.0)	
	Covered/no surgery	106 (91.4)	81 (63.3)		173 (77.9)	14 (63.6)	
**Laboratory tests cost**	<150 USD/session	36 (31.0)	47 (36.7)	0.349	78 (35.1)	5 (22.7)	**0.024**
	≥150 USD/session	80 (69.0)	81 (63.3)		144 (64.9)	17 (77.3)	
**NCCN compliance**	Non-compliant	11 (9.48)	26 (20.3)	**0.019**	20 (9.0)	17 (77.3)	**<0.001**
	Compliant	105 (90.5)	102 (79.7)		202 (91.0)	5 (22.7)	

The percentages are presented as row percentages. Bold *p*-values indicate statistical significance (*p* < 0.05). * Borderline significance. NCCN = National Comprehensive Cancer Network.

**Table 4:** Multivariable logistic regression identifying independent predictors of delayed treatment initiation.

Variable	Category	aOR	95% CI	*p*-Value
**Age**	per year increase	1.01	0.99–1.03	0.203
**Sex**	Female (ref.)	1.00	—	—
	Male	1.26	0.70–2.29	0.432
**Educational level**	<High school (ref.)	1.00	—	—
	High school or higher	0.82	0.44–1.52	0.545
**Occupation**	Employed/retired (ref.)	1.00	—	—
	Unemployed/student	0.55	0.29–1.03	0.062
**Household income source**	Fixed (ref.)	1.00	—	—
	Unfixed	2.55	1.42–4.59	**0.002**
**Medical insurance**	Insured (ref.)	1.00	—	—
	Uninsured	1.91	1.08–3.39	**0.026**
**Pre-treatment surgical cost**	Covered/No surgery (ref.)	1.00	—	—
	≥1000 USD	9.03	2.57–31.7	**0.001**

Reference category (OR = 1.00, —). Bold values in the text indicate statistical significance (*p* < 0.05).

**Table 5:** Multivariable logistic regression identifying independent predictors of incomplete treatment.

Variable	Category	aOR	95% CI	*p*-Value
**Sex**	Female (ref.)	1.00	—	—
	Male	0.54	0.18–1.62	0.273
**Educational level**	<High school (ref.)	1.00	—	—
	High school or higher	0.33	0.10–1.07	0.056 *
**Occupation**	Employed/retired (ref.)	1.00	—	—
	Unemployed/student	1.14	0.34–3.85	0.836
**Household income source**	Fixed (ref.)	1.00	—	—
	Unfixed	2.09	1.16–5.79	**0.015**
**Medical insurance**	Insured (ref.)	1.00	—	—
	Uninsured	5.62	1.22–25.9	**0.027**
**Time since diagnosis**	≤1 year (ref.)	1.00	—	—
	>1 year	0.26	0.03–2.14	0.210
**Laboratory tests cost/session**	<USD 150 (ref.)	1.00	—	—
	≥USD 150	1.62	1.02–2.57	**0.041**

Reference category (OR = 1.00, —). Bold values in the text indicate statistical significance (*p* < 0.05). * Borderline significance.

## Data Availability

The original contributions presented in this study are included in the article. Further inquiries can be directed to the corresponding author.

## Abbreviations

The following abbreviations are used in this manuscript:

IARC	International Agency for Research on Cancer
LMICs	Low- and middle-income countries
MENA	Middle East and North Africa
QOL	Quality of life
GDP	Gross domestic product
MOPH	Ministry of Public Health
USD	U.S. dollars
LBP	Lebanese pounds
NCCN	National Comprehensive Cancer Network
aORs	Adjusted odds ratios
CIs	Confidence intervals
IRB	Institutional Review Board
